# Effect of benralizumab on histopathology and inflammatory signatures in a clinical cohort of eosinophilic esophagitis

**DOI:** 10.1093/dote/doae031

**Published:** 2024-07-11

**Authors:** Ashley L Pyne, Amiko M Uchida, Mark W Hazel, Chris J Stubben, Joy W Chang, Dominique D Bailey, Nirmala Gonsalves, Kristina Allen-Brady, Kathryn A Peterson, Maria A Pletneva

**Affiliations:** Division of Gastroenterology, Hepatology & Nutrition, University of Utah School of Medicine, Salt Lake City, UT, USA; Division of Gastroenterology, Hepatology & Nutrition, University of Utah School of Medicine, Salt Lake City, UT, USA; Division of Gastroenterology, Hepatology & Nutrition, University of Utah School of Medicine, Salt Lake City, UT, USA; Cancer Bioinformatics Resource, Huntsman Cancer Institute, University of Utah, Salt Lake City, UT, USA; Division of Gastroenterology, Department of Internal Medicine, University of Michigan, Ann Arbor, MI, USA; Division of Pediatric Gastroenterology, Hepatology, and Nutrition, Department of Pediatrics, Columbia University Vagelos College of Physicians & Surgeons and NewYork-Presbyterian Morgan Stanley Children's Hospital, New York, NY, USA; Division of Gastroenterology & Hepatology, Northwestern University – Feinberg School of Medicine, Chicago, IL, USA; Division of Epidemiology, Department of Internal Medicine, University of Utah, Salt Lake City, UT, USA; Division of Gastroenterology, Hepatology & Nutrition, University of Utah School of Medicine, Salt Lake City, UT, USA; Department of Pathology, University of Utah School of Medicine, Salt Lake City, UT, USA

**Keywords:** Allergy, transcriptome, IL-5 alpha receptor alpha, Histology Scoring System, RNA sequencing

## Abstract

A preliminary report from the recent phase 3 trial of benralizumab, a monoclonal antibody that binds to interleukin-5 receptor alpha (IL5Rα), in patients with EoE revealed that medication use led to tissue eosinophil eradication but did not meet the clinical endpoint of symptom resolution. Here, we characterized the clinical, endoscopic, histologic, and transcriptional changes in patients with active EoE following benralizumab treatment. We retrospectively examined patients with EoE treated with benralizumab at the University of Utah (*n =* 11) and reviewed reported clinical symptoms, circulating and tissue eosinophilia, and endoscopic and histologic scores. Gene expression profiles from available esophageal tissue from benralizumab-treated patients were compared to those from patients with remission EoE (*n =* 5), active EoE (*n =* 10), and controls (*n =* 22). Benralizumab treatment resulted in partial symptom improvement and significant reduction in tissue eosinophilia, and endoscopic and histologic disease scoring (*P* < 0.01). Histologic score reductions were driven by eosinophil feature scores, while scores for epithelial features (basal cell hyperplasia and dilated intercellular spaces) were similar to those in active EoE. The gene signatures in benralizumab-treated patients mimicked those of active EoE (e.g. upregulation of *POSTN, CDH26, CCL26,* and downregulation of *DSG1*). RNA profiles and pathways support histologic findings of impaired epithelial function that persists despite benralizumab treatment. In conclusion, despite eosinophil eradication, patients treated with benralizumab had persistent epithelial injury at the histologic and transcriptional level. In this cohort, benralizumab therapy failed to eradicate inflammation and epithelial dysfunction showing that interleukin-5 receptor alpha blockade monotherapy is insufficient to control EoE.

## INTRODUCTION

Eosinophilic gastrointestinal diseases (EGIDs) are so named due to their characteristic gastrointestinal mucosal eosinophilic infiltration easily identified on routine hematoxylin and eosin (H&E) staining of tissue. Eosinophilic esophagitis (EoE) is the most common EGID and is increasing in incidence and prevalence.^1^ Eosinophilic inflammation in EoE is driven by type 2 inflammation with cytokines such as interleukin (IL)-4, IL-5, and IL-13.^2^ Therapeutic options are tailored to the site of involvement and include topical or systemic corticosteroids, proton pump inhibitors (PPIs), and elimination diets.^3^^,^^4^ There is considerable interest in eosinophil-targeting biologic therapies that have efficacy in other eosinophil-related diseases, such as eosinophilic asthma or other EGIDs. To date, dupilumab, targeting the IL-4 receptor alpha subunit, is the only United States Food and Drug Administration (FDA)-approved biologic for EoE treatment.^5–7^

Benralizumab is a cytolytic monoclonal IgG1 antibody directed against the IL-5 receptor alpha (IL-5Rα) expressed on eosinophils and basophils.^8^^,^^9^ It inhibits the binding of IL-5 as well as the hetero-oligomerization of the alpha and beta subunits of the IL-5R, thus blocking signal transduction. IL-5 is a key cytokine in the survival and persistence of circulating and tissue eosinophils.^10^ Benralizumab is indicated for add-on maintenance care of asthma in patients with peripheral eosinophil counts of 300k/μL or more.^11^ A Phase 3 study investigating the use of benralizumab as a treatment for patients with EoE was recently completed (ClinicalTrials.gov identifier: NCT04543409) and reported a lack of symptomatic efficacy endpoint despite the resolution of circulating and tissue eosinophilia.^12^ This persistence of non-eosinophilic disease features warrants further investigation into the mechanisms involved in a disease thought to be driven by eosinophils.

We retrospectively evaluated the effects of benralizumab in adults with active EoE who failed standard-of-care therapies and received benralizumab. We describe benralizumab-mediated changes to clinical, endoscopic, and histologic features of the esophagus. We also evaluated the esophageal transcriptional profile following benralizumab treatment compared to patients with active and inactive EoE, and non-EoE controls. Collectively, our data provide insight into the IL-5Rα-mediated effects in a multi-inflammatory pathway disease.

## METHODS

### Patient cohort

We conducted a retrospective study on benralizumab-treated patients with EoE from 2018 to 2021 at the University of Utah clinics (IRB approval #89836). We identified 11 patients who received benralizumab as part of clinical care via gastroenterology or allergy clinic (via insurance coverage for compassionate use, FDA-approved comorbidity, or as a sample). All patients were given benralizumab initially in an effort to deplete eosinophils after failing multiple standard-of-care therapies for EoE; none of these patients were involved in the industry-sponsored MESSINA clinical trial. Patients were given benralizumab (30 mg subcutaneously) for a minimum of 3 months with injections every 8 weeks after the initial three induction doses per standard asthma therapy. Follow-up esophagogastroduodenoscopy (EGD) did not follow a standard protocol and was utilized as clinical care dictated, occurring as soon as 2 weeks to as late as 8 months after benralizumab initiation. Prior to starting benralizumab, all patients met consensus criteria for active EoE with peak esophageal tissue eosinophil count of ≥15 eosinophils per high power field (eos/HPF) and esophageal symptoms.^13^ Esophageal formalin-fixed, paraffin-embedded tissue was available from 6 of the 11 patients to assess gene expression following benralizumab treatment, an average of 2.65 months post first injection (Supplemental Table 1). Similarly prepared and sequenced esophageal tissue from patients with EoE in remission induced by diet (*n =* 5), active EoE (*n =* 10), and non-EoE controls (*n =* 22) were used for gene expression comparisons. All non-EoE controls had normal histology and no eosinophils. Demographics, diagnoses, history of EoE-related therapies, esophageal tissue eosinophil counts, peripheral eosinophil counts, EoE Endoscopic Reference Scores (EREFS), and clinical symptoms were documented prior to and following benralizumab treatment, when possible.

### Clinical symptoms, endoscopic, and histologic outcomes

Patient-reported clinical symptoms were subjective and based on physician-reported conversations with the patients during clinical visits. EREFS scores were evaluated by the same endoscopist either at the time of procedure or based on endoscopic images performed by another provider. Histologic parameters were evaluated by a single gastrointestinal pathologist with expertise in EGID. To evaluate the severity and extent of EoE, EoE Histology Scoring System (EoEHSS)^14^ was used on available esophageal tissue (e.g. proximal, mid, and/or distal) from patients pre- and post-benralizumab treatment, 5 patients with remission EoE, and 10 patients with active EoE. The pathologist scored grade and stage on esophageal inflammation (EI), basal zone hyperplasia (BZH), eosinophil abscess (EA), eosinophil surface layering (ESL), dilated intercellular spaces (DIS), surface epithelial alteration (SEA), dyskeratotic epithelial cells (DECs), and lamina propria fibrosis (LPF), when applicable. A normalized composite score ratio was calculated to account for any missing features (i.e. lamina propria) and designated as composite score as previously described.^15^

### RNA isolation, sequencing, and genome alignment

Total RNA from esophageal biopsies was isolated from five scrolls per sample of 10-micron, formalin-fixed paraffin-embedded tissue with the High Pure FFPE RNA isolation kit (Roche Diagnostics) that includes on-column DNase digestion. RNA yield averaged 5.2 μg/sample and was determined with a NanoDrop-1000 spectrophotometer (Thermo Scientific). Prior to sequencing, RNA ScreenTape Assay with an Agilent 2200 TapeStation was performed to assess RNA quality; all samples had 60% or more RNA between 200 and 5000 nt. Stranded RNA libraries were prepared with the Illumina TruSeq RNA Exome library kit, and samples were sequenced on an Illumina NovaSeq 6000 instrument using 150-bp paired-end reads. Reads within the fastq files were aligned to the GRCh38 human reference genome using the STAR aligner and Ensembl version 102 reference. Human-aligned reads per sample averaged 26.8 million.

### Differential expression and gene set enrichment analyses

Differentially expressed genes were identified using DESeq2^16^ version 1.36.0 with a Benjamini–Hochberg adjustment for multiple testing and a false discovery rate (FDR) cut-off of 0.05. Genes were defined as differentially expressed across patient categories if they differed with an adjusted *P*-value of <0.05. The DESeq2 analysis controlled for sex-related RNA differences by modelling disease and sex in the design formula. The shrunken log2 fold changes from DESeq2 were used for gene set enrichment analysis (GSEA) using the fgsea package.^17^ Hallmark gene sets were employed to identify pathways significantly enriched with up- or down-regulated genes. Pathways with a 10% FDR were selected for comparison between sample categories. A linear mixed-effects model of variance was conducted with the variancePartition software,^18^ using the variance stabilizing transformed counts from DESeq2 and metadata of sex, age, patient category, peak eos/HPF, fibrotic EREFS score, inflammatory EREFS score, and overall HSS stage score. Overall HSS grade score was excluded from the linear mixed effect model as it was highly correlative with the overall HSS stage score.

### Statistical analysis

Patient demographics were compared among patient categories using Mann–Whitney (or Kruskal–Wallis with Dunn’s multiple comparison test) based on the number of groups compared (GraphPad Prism v10.0.0). Paired benralizumab treatment analysis of eos/HPF, EREFS scores, and EoEHSS scores used Wilcoxon matched-pairs signed-rank test. EoEHSS composite and feature scores were compared among groups using Welch’s ANOVA to account for standard deviations variation. EoEHSS grade and severity scores were averaged if more than one esophageal biopsy location was available.

## RESULTS

### Clinical characteristics

Of the 11 EoE patients receiving benralizumab, four patients had concurrent diagnoses of eosinophilic gastritis and duodenitis, of which one patient was subsequently diagnosed with eosinophilic granulomatosis with polyangiitis, and lastly, one patient had concurrent eosinophilic colitis (Table 1). EoEHSS grade and stage composite scores did not differ between patients with EoE only and those with concurrent distal involvement (*P* > 0.99). All patients failed multiple EoE therapies including topical corticosteroids, systemic steroids, and elimination diets with persistent symptoms and esophageal eosinophilia ≥15 eos/HPF prior to initiating benralizumab. However, before benralizumab treatment, patients’ EoE disease severity was not statistically different than the active EoE comparison group for peak eosinophil counts (*P* = 0.30), EREFS (*P* = 0.28), or EoEHSS grade composite score (*P* = 0.27). Additionally, 82% of patients complained of extra-esophageal symptoms including abdominal pain (82%), food intolerances (36%), and nausea (36%). One patient had complications during follow-up EGD, and biopsies were not taken; therefore, this individual was excluded from paired tissue–based analyses. There was no significant difference between the age or sex ratios among EoE comparison groups.

**Table 1:** Patient demographics and clinical characteristics of patient prior to and following benralizumab treatment

	Benralizumab active EoE (*n* = 11)	Active EoE (*n* = 10)	Remission EoE (*n* = 5)	Non-EoE controls (*n* = 22)
Age (mean, range)	35.6 (19–71)	33.2 (21–44)	27.4 (19–34)	38.5 (19–69)
Male (%)	54.5%	60.0%	60.0%	36.4%
Peak tissue eos/HPF (mean, range)	105 (23–252)	47.8 (15–100)	2.6 (0–7)	0 (0–0)
Endoscopy Reference Score (mean EREFS sum)	3.09	2.6	0	n/a
EoE Histology Scoring System (mean composite score)	Grade: 0.49 Stage: 0.48	0.38 0.28	0.09 0.06	n/a
Organs involved (%)	36.4 %—multiple GI segment involvement	10.0%—multiple GI segment involvement	0%—multiple GI segment involvement	n/a
Stomach	4	1	0	
Duodenum	4	0	0	
Colon	1	0	0	
	**Prior to benralizumab (n = 11)**		**Following benralizumab (n = 11)**	
SOC therapies				
PPI	100% (11)		90.9% (10)	
Topical steroids	90.9% (10)		0.0% (0)	
Systemic steroids	81.8% (9)		27.3% (3)	
Biologic	18.2% (2)		9.1% (1)	
Dietary therapy	100.0% (11)		27.3% (3)	
Symptoms	100.0% (11)		72.7% (7)	
Dysphagia	100.0% (11)		36.4% (4)	
Nausea	36.4% (4)		18.2% (2)	
Abdominal pain	81.8% (9)		54.5% (6)	
Food intolerance	36.4% (4)		27.3% (3)	
Peak tissue eos/HPF (mean, range)	105 (23–252)		0.3 (0–3)	
Endoscopy Reference Score (mean EREFS sum)	3.09		1.18	
EoE Histology Scoring System (mean composite score)	Grade: 0.49 Stage: 0.48		0.28 0.24	
Peripheral eosinophils counts (mean k/μL)	0.53		<0.03	

EoE, eosinophilic esophagitis; eos/HPF, eosinophils/high power field; n/a, not applicable; PPI, proton-pump inhibitor; SOC, standard of care.

### Benralizumab therapy response

Follow-up EGD after the first benralizumab infusion averaged 2.65 months (Supplemental Table 1; Supplemental Fig. 1). Eight of 11 patients reported improvement in at least one symptom following administration of benralizumab, most commonly citing resolution of dysphagia or abdominal pain (Table 1). Adverse reactions following benralizumab included moderate chest and throat tightness (patient ID# 6) and increased abdominal pain (patient ID# 10).

**Fig. 1:**
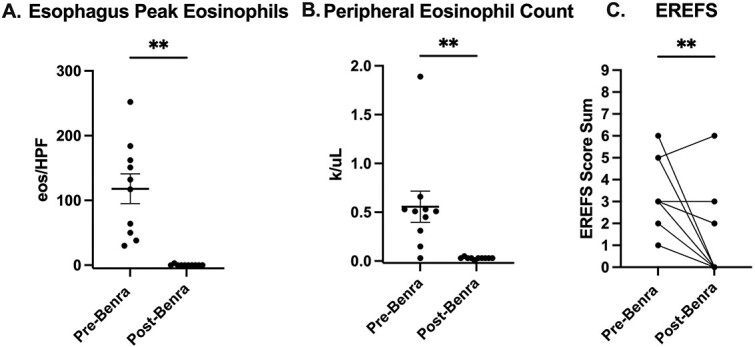
Benralizumab significantly improves eosinophilic and endoscopic response in patients with baseline active eosinophilic esophagitis (EoE). Paired before benralizumab (pre-Benra) and following benralizumab treatment (post-Benra) patient samples. (A) Peak esophageal eosinophil counts displayed in eosinophils per high power field (eos/HPF) (n=10). (B) peripheral blood eosinophil counts (k/μL) (n=10). (C) endoscopic reference scoring (EREFS) shown as a sum of each clinical endoscopy findings score (n=11). Wilcoxon matched-pairs signed rank test, mean + SEM; ^*^^*^^*^ p<0.001, ^*^^*^ p<0.01, ^*^p<0.05.

All patients demonstrated near-complete eradication of eosinophils from esophageal tissue. Blood eosinophil counts were also depleted over the dosing period (Table 1 and Fig. 1). In patients with distal involvement, the stomach and duodenum also demonstrated eosinophil depletion (stomach average 188 eos/HPF down to 0.3 eos/HPF; duodenum average 80 eos/HPF down to 0.7 eos/HPF), in agreement with recent phase 2 findings^19^ (Supplemental Fig. 2). EREFS scores also significantly improved in 9 of 11 patients following benralizumab (Fig. 1).

**Fig. 2:**
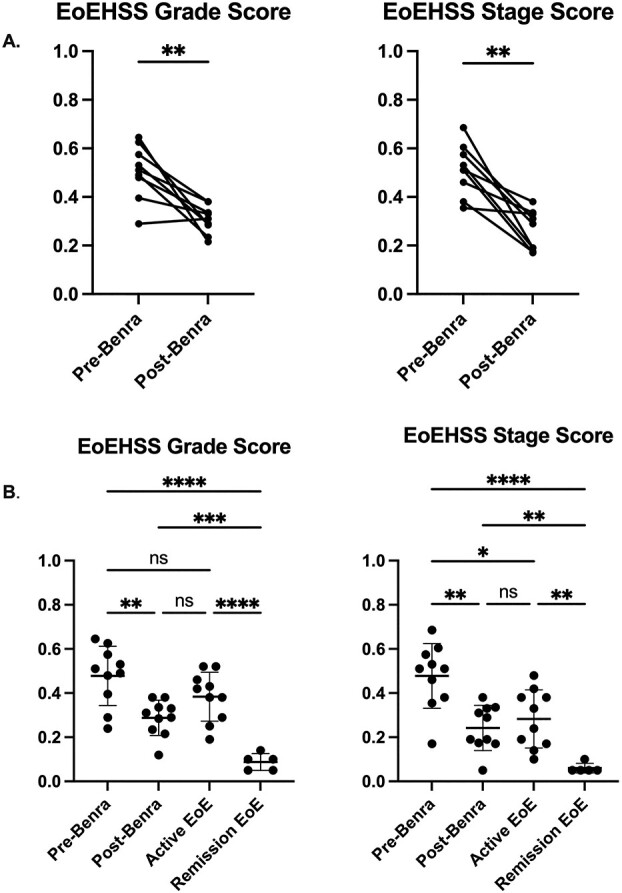
Benralizumab improved histologic severity and extent among treated eosinophilic esophagitis patients, but histologic improvement following benralizumab treatment was comparable to active disease. Showing composite grade and stage score, grade score closer to “1” indicates more severe EoE. Similarly, a stage score closer to “1” indicates more extensive disease. (A) Composite EoE histologic scores in paired patients before (pre) and following (post) benralizumab treatment (n=9), Wilcoxon matched-pairs signed-rank test. (B) Composite EoE histologic scores in patients before and following benralizumab treatment (n=10), with active EoE (n=10), with remission EoE (n=5), Welch’s ANOVA, mean + SD. ^*^^*^^*^^*^ p<0.0001, ^*^^*^^*^ p<0.001, ^*^^*^ p<0.01, ^*^p<0.05, non-significant (ns).

**Fig. 3:**
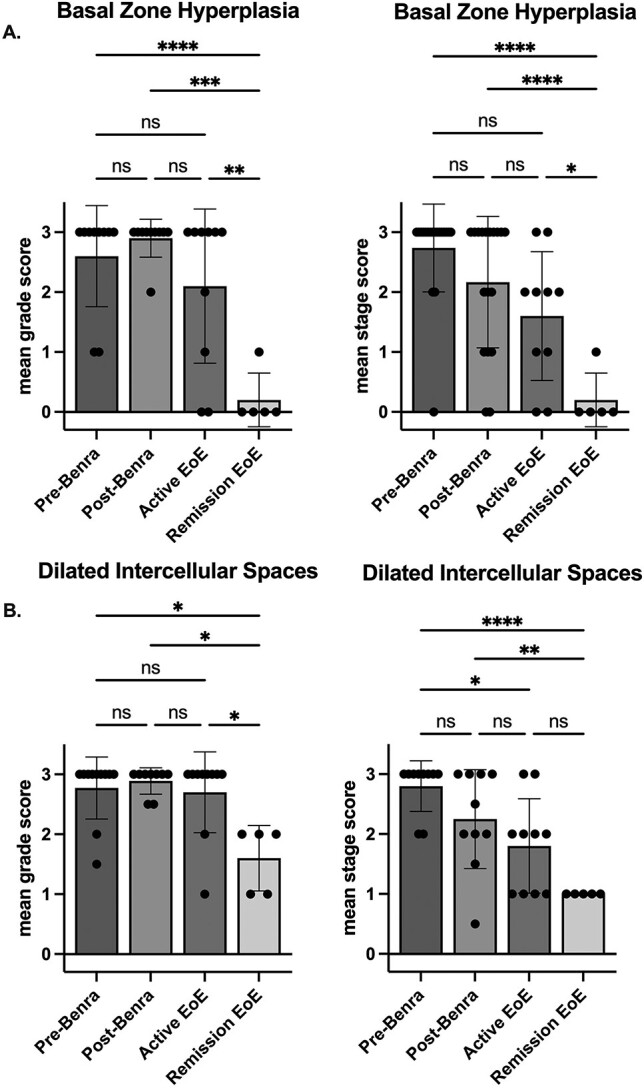
Average eosinophilic esophagitis histologic scoring system grade and stage scores for basal zone hyperplasia and dilated intercellular spaces features. Grade (severity) and stage (extent) scale is 0 to 3. (A) basal zone hyperplasia for grade and stage for patients before and following benralizumab (n=10), with active EoE (n=10) and remission EoE (n=5) (B) dilated intercellular spaces with same patient group comparison. Welch’s ANOVA, mean + SD. ^*^^*^^*^^*^ p<0.0001, ^*^^*^^*^ p<0.001, ^*^^*^ p<0.01, ^*^p<0.05, non-significant (ns).

Histopathologic improvement was seen as soon as 10 days following the first dose of benralizumab (Supplemental Table 1). HSS grade and stage composite scores significantly decreased following the start of benralizumab (Table 1 and Fig. 2a). Benralizumab infusion had the greatest effect on eosinophil markers, with improved grade and stage scores for EI, EA, and ESL (Table 2). Epithelial injury parameters, such as BZH, DIS, and LPF, had little to no change following benralizumab therapy. Despite the reduction of esophageal eosinophils to the same degree as in patients who achieved remission via an elimination diet, benralizumab patients had significantly higher HSS grade score. Moreover, HSS grade and stage scores were not significantly different between benralizumab-treated patients and active EoE (Fig. 2b), with no difference in BZH and DIS grade and stage scores (Fig. 3a and b). Lamina propria fibrosis could not be compared due to inadequate sample size.

**Table 2:** Eosinophilic esophagitis histology scoring system in patients before and following benralizumab treatment

Patient ID	Benralizumab status	Esophagus location	Peak eos/HPF	^*^Grade score	^†^Stage score	EI grade	EI stage	BZH grade	BZH stage	EA grade	EA stage	ESL grade	ESL stage	DIS grade	DIS stage	SEA grade	SEA stage	DEC grade	DEC stage	LPF grade	LPF stage
1	Pre	Distal	23	0.24	0.1	2	1	0	0	0	0	0	0	3	1	0	0	0	0	n/a	n/a
2	Pre	Distal	162	0.79	0.67	3	3	3	3	3	1	1	1	3	3	2	1	1	1	3	3
	Post	Distal	0	0.33	0.33	0	0	0	3	3	0	0	0	0	3	3	1	1	0	1	3
3	Pre	Proximal	117	0.58	0.63	3	3	3	3	1	1	1	1	3	3	0	0	1	1	2	3
	Post	Proximal	0	0.38	0.25	0	0	3	3	0	0	0	0	3	2	0	0	0	0	3	1
4	Pre	Esophagus	30	0.29	0.38	2	3	1	2	0	0	0	0	3	3	0	0	0	0	n/a	n/a
	Post	Distal	0	0.33	0.21	0	0	3	2	0	0	0	0	3	2	0	0	0	0	2	1
5	Pre	Distal	252	0.52	0.52	3	3	3	3	1	1	1	1	3	3	0	0	0	0	n/a	n/a
	Post	Distal	0	0.38	0.38	0	0	3	3	0	0	0	0	3	3	0	0	0	0	3	3
6	Pre	Proximal	151	0.79	0.79	3	3	3	3	3	1	1	3	3	3	3	3	0	0	3	3
	Post	Proximal	0	0.1	0.05	0	0	0	0	0	0	0	0	2	1	0	0	0	0	n/a	n/a
7	Pre	Distal	38	0.46	0.38	2	1	3	3	0	0	0	0	3	3	0	0	0	0	3	2
	Post	Distal	0	0.33	0.33	0	0	3	3	0	0	0	0	3	3	1	1	0	0	n/a	n/a
8	Pre	Proximal	184	0.48	0.52	3	3	3	3	1	2	0	0	3	3	0	0	0	0	n/a	n/a
	Post	Proximal	0	0.33	0.24	0	0	3	2	0	0	0	0	3	2	1	1	0	0	n/a	n/a
9	Pre	Distal	64	0.5	0.5	3	3	3	3	0	0	0	0	3	3	0	0	0	0	3	3
	Post	Distal	3	0.29	0.29	0	0	3	3	0	0	0	0	3	3	0	0	0	0	n/a	n/a
10	Post	Distal	0	0.24	0.1	0	0	2	1	0	0	0	0	3	1	0	0	0	0	n/a	n/a
11	Pre	Proximal	132	0.54	0.54	3	3	3	3	1	1	1	2	3	3	0	0	0	0	2	1
	Post	Distal	0	0.29	0.19	0	0	3	2	0	0	0	0	3	2	0	0	0	0	n/a	n/a

Patients before (pre) and following (post) benralizumab treatment in tissue location with peak eosinophil counts and associated histology scores. Patient 1 had no tissue collected during follow-up due to complications during EGD. Patient 10 tissue was unavailable as EGD was completed outside of university system. To account for missing features, scores were normalized by dividing the observed total score by the maximal possible score for that biopsy (e.g. maximum score 21 for those missing lamina propria) to result in a ‘total score ratio’ that is designated as ‘composite score’ in the table.^14^

^*^Composite histology grade scoring is severity; scale 0 (normal) to 3 (severe).

^†^Composite histology stage scoring is extent; scale 0 (normal) to 3 (severe). BZH, basal zone hyperplasia; DIS, dilated intercellular spaces; DEC, dyskeratotic epithelial cells; EA, eosinophil abscess; ESL, eosinophil surface layering; eos/HPF, eosinophils/high power field; EI, esophageal inflammation; LPF, lamina propria fibrosis; n/a, not available; SEA, surface epithelial alteration.

### Benralizumab-treated esophageal transcriptional signatures

**Fig. 4:**
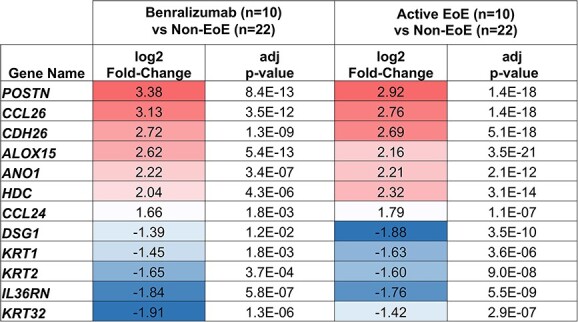
Genes known to be associated with active eosinophilic esophagitis. Selected genes known to be associated with active EoE from prior publications with benralizumab treatment patients compared to non-EoE controls (left) and patients with active EoE compared to non-EoE controls (right). Red indicates upregulated genes in benralizumab treated or active EoE with positive log2 fold-change/fold-change and blue indicates negative log2 fold-change/fold-change.

We performed comparisons of esophageal tissue RNA sequencing results from patients following the treatment with benralizumab (*n =* 6) to controls (*n =* 22) and identified 4113 differentially expressed genes (DEGs). Benralizumab-treated patients had transcriptional signatures related to impaired barrier function and inflammation characteristic of active EoE when compared to controls. Benralizumab-treated patients had higher RNA expression levels for periostin (*POSTN*), eotaxin-3 (*CCL26*), and cadherin-26 (*CDH26*) and lower RNA expression levels for desmoglein 1 (*DSG1*). Interestingly, benralizumab-treated patients had similar fold-changes and adjusted *P*-values for these EoE-associated genes as active EoE patients when compared to controls (Fig. 4).

 When compared to patients inducing EoE remission by diet (*n =* 5; average diet intervention duration 1.7 months), benralizumab-treated patients had 1595 DEGs. Factors regulating tissue remodeling (*POSTN*, Keratin 14 [*KRT14*], anoctamin 1 [*ANO1*]), and eosinophilic infiltration (interleukin 13 receptor subunit alpha 2 [*IL13RA2*] and *CCL26*) continued to be elevated in benralizumab-treated patients. In correlation with the DEGs results, pathways showed that benralizumab-treated patients had upregulated genes in inflammatory pathways such as IL6 Jak Stat3 Signaling, IL2 Stat5 Signaling, Complement, Interferon Alpha Response, and Interferon Gamma Response with a normalized enrichment score (NES) greater than 1.4 when compared to remission EoE and controls (Fig. 4). Interestingly, the Notch Signaling pathway, critical in squamous epithelial homeostasis, was significantly downregulated (NES −1.5) in benralizumab-treated patients.

**Fig. 5:**
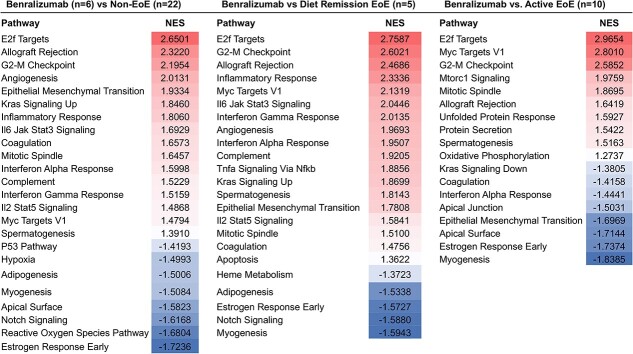
Benralizumab treated patients have up-regulated genes in inflammatory pathways and down-regulated genes in tight junction pathways despite eosinophil depletion. Gene set enrichment analysis using Hallmark gene sets from benralizumab treated compared to non-eosinophilic esophagitis (EoE) controls, EoE remission induced by diet, and Active EoE. Significant pathway differences (10% FDR) with the highest and lowest normalized enrichment score (NES) are shown.

Finally, we compared benralizumab-treated patients to active EoE and identified 2254 DEGs (1238 upregulated, 1016 downregulated). Benralizumab tissues had higher RNA levels for factors involved in T cell proliferation and differentiation (i.e. IL-7 receptor [*IL7R*], IL-2 receptor subunit alpha [*IL2RA*], and chemokine ligand 20 [*CCL20*]) compared to active EoE. However, following benralizumab treatment, there were no inflammatory-associated GSEA pathways upregulated when compared to active EoE (Fig. 5). Epithelial integrity pathways were significantly downregulated when compared to patients with active EoE: apical surface (NES −1.7), apical junction (NES −1.5), and epithelial–mesenchymal transition (NES −1.7).

To explore contributions to variance in gene expression from disease severity parameters, we fit a linear mixed-effect model using the variancePartition package. A linear mixed-effect model identifies genes where a large fraction of variation in expression was explained by a single disease severity variable. HSS staging score explained the largest fraction of the variance (17.3%) followed by presence of disease activity and treatment (8.4%) (i.e. patient category) (Supplemental Fig. 3). EREFS scores accounted for a smaller fraction of the variance (fibrotic EREFS 6.4% and inflammatory EREFS 4.4%), while peak eosinophil count and age had a small effect (3%, respectively). IL-18 was one of the genes with a large fraction of variation from HSS stage score. It is postulated to be part of a type 2 inflammatory response via its influence on IL-5 and IL-13 cytokine production.^20^ Several other genes associated only with HSS stage score are found to be expressed in CD4+ T cells and impact the differentiation and effector function, such as adenosine deaminase (*ADA*), vitamin D receptor (*VDR*), and calcium binding protein 39 (*CAB39*).^21–23^

## DISCUSSION

Here, we described a cohort of patients with severe EoE treated with benralizumab and identified clinical, histologic, and transcriptional changes following treatment. We found that benralizumab significantly improved EREFS scores, EoEHSS, and eosinophil counts. However, eosinophil-independent inflammation was not histologically improved (i.e. BZH, DIS). Esophageal transcriptional signatures in benralizumab-treated patients displayed ongoing epithelial injury and inflammation that were more similar to active than inactive EoE. Collectively, our results demonstrate that targeting IL-5Rα is insufficient to ameliorate the epithelial and non-eosinophil inflammatory injury that is characteristic of EoE.

Our findings are consistent with clinical trials evaluating IL-5-specific targeting in EoE where eosinophil depletion was noted but with only partial improvement in esophageal symptoms.^12^^,^^24^ IL-5 is a critical factor for promoting eosinophil development, activation, survival, and recruitment. Therefore, targeting it or the IL-5 receptor is highly specific for eosinophils.^25^ While targeting of IL-5 or its receptor is an effective, additive therapy in eosinophilic asthma, clinical trials and our current study suggest that isolated blockade of IL-5 signaling is insufficient to capture the entirety of the inflammatory cascade in EoE.^24^^,^^26^ Alterations following benralizumab treatment did not appear to be related to the duration of therapy, since the average time between the first dose of benralizumab and EGD follow-up was 2.6 months, whereas the follow-up of patients managed with diet was shorter at 1.7 months. The higher BZH and DIS scores seen in benralizumab-treated patients compared to patients who induced remission with diet suggest that benralizumab-mediated eosinophil depletion does not entirely repair epithelial damage in EoE that could arise from antigenic exposure (e.g. eating a food trigger). This suggests that eosinophils may be downstream of more proximal inflammatory signals and may not be the only mediators of injury in this disease.

Our differential expression results support histologic observations that inflammation remains in the esophageal epithelium despite eosinophil eradication. We found the transcriptional signature of benralizumab-treated patients to be more similar to that of active EoE patients than remission EoE or control patients. Eotaxin RNA levels of CCL26 and CCL24, which are potent eosinophil chemoattractants induced by IL-13 and IL-4, were increased in benralizumab-treated patients relative to controls and remission EoE patients. These findings suggest that blockade of IL-5 receptor signaling is insufficient to abrogate other type 2 inflammatory cytokines such as IL-4 and IL-13. Additionally, transcripts for histidine decarboxylase (*HDC*) were elevated. In the esophageal mucosa, the most relevant cells capable of HDC production are mast cells, suggesting their involvement in sustained epithelial injury after benralizumab therapy. This hypothesis is supported by GSEA pathway results of upregulated genes in adaptive immunity and cytokine receptor inflammatory pathways: interferon alpha response, interferon gamma response, inflammatory response, and complement.

The dysregulation that promotes loss of epithelial integrity in benralizumab-treated patients hints at a possible mechanism of sustained disease driven by IL-13 and/or IL-4. With increased levels in *POSTN* and *ANO1* and reduced levels of *DSG*-*1* when compared to controls and remission EoE, we hypothesize IL-13 as one of the driving factors in epithelial barrier disruption. *POSTN* is a cell adhesion factor secreted primarily from esophageal epithelial cells when stimulated by IL-13.^27^*ANO1* is also stimulated by IL-13 and was elevated in active EoE specimens and correlated with disease severity. Importantly, ANO1 is expressed by esophageal basal cells and is thought to play a role in hyperplasia.^28^ Lastly, we saw a lower level of *DSG-1*, known to be downregulated by IL-13. GSEA pathway results suggest a role for IL-4. The JAK/STAT pathway is important for cytokine signaling and in the development of fibrosis.^29^ We found an upregulation of multiple genes in the IL-2 Jak Stat3 and IL-6 Jak Stat3 pathways in benralizumab-treated patients. Interestingly, IL-2 and IL-6 are associated with promotion of IL-4.^20^^,^^30^^,^^31^

*In vivo* biologic trials have been valuable to better understand EGID pathophysiology.^32^ Our results warrant further investigation of the transcriptional differences between treatment with benralizumab and active EoE with a larger sample size to elucidate mechanism underlying EoE. Patients on benralizumab appear to have dysregulation of genes and pathways related to T cell proliferation and differentiation, and epithelial function. Interestingly, the epithelial–mesenchymal transition pathway was downregulated in benralizumab-treated patients compared to patients with active EoE. The epithelial–mesenchymal transition has been associated with the activation of myofibroblasts, suggesting blocking IL-5 could play a protective role against fibrogenesis in EoE.^33^

A strength of this study is its multidimensional assessment of benralizumab therapy in a real-life clinical setting combining clinical observations, histopathology, and RNA sequencing, showing for the first time transcriptional signatures in patients treated with benralizumab. Our study results were similar to initial reports from the phase 3 clinical study indicating that benralizumab improved disease scoring largely by ameliorating eosinophil-related disease factors, but only modestly improved clinical outcomes. Despite these strengths, there were limitations to our study. Follow-up intervals were variable. However, we found similar eosinophil reduction and EoEHSS stage and grade scores following benralizumab treatment across a follow-up interval spectrum from less than 2 months to 8 months. Limited availability of paired tissue samples is reflective of standard clinical care challenges, and its possible impact on transcriptional read-out is a major limitation of this study. However, our transcriptional comparison of benralizumab-treated patients to controls and remission EoE spotlight a broader type 2 inflammatory milieu beyond IL-5, including likely important roles for IL-4 and IL-13, as blockade of these with dupilumab is the only study to date to show any histologic and symptomatic improvement.^7^

Benralizumab is effective in eosinophil depletion and some improvement in symptoms in our cohort, but our results indicate ongoing components of inflammation and epithelial barrier dysfunction both histologically and transcriptionally. The persistence of epithelial injury indicates the activity of eosinophil-independent mechanisms of disease and adds fuel to the ongoing debate about the role of eosinophils in EoE pathobiology. Our RNA sequencing data demonstrate differential expression of genes associated with IL-13- and IL-4-related inflammatory pathways and mast cells, suggesting their continued role in the disease process despite the absence of eosinophils. We therefore hypothesize that a multifaceted regulator, such as STAT6, a known regulator of IL-13 in EoE, could also perpetuate IL-13 and IL-4 functions outside of eosinophils, such as modulation of mast cells, B cell activation, or fibroblast proliferation. Such functions could contribute to the persistent inflammatory and epithelial injury features seen following benralizumab treatment. However, this hypothesis remains to be tested in further studies. Our current study leverages clinical specimens and data to add incremental insights into the role of type 2 inflammation in eosinophilic esophageal disease. Furthermore, our findings demonstrate that targeting IL-5Rα signaling in isolation is insufficient to capture the entirety of EoE inflammation, adding to the growing evidence that this complex disease cannot be attributed to a single cell type.

## Supplementary Material

Benralizumab_and_EoE_Supplemental_Figures_doae031

Benralizumab_Manuscript_Large_Tables_Revision1_doae031

## ABBREVIATIONS

*ADA*, adenosine deaminase; *ANO1*, anoctamin 1; BZH, basal zone hyperplasia; *CAB39*, calcium binding protein 39; *CCL20*, chemokine ligand 20; *CCL26,* eotaxin-3; *CDH26,* cadherin-26; DEC, dyskeratotic epithelial cells; DEGs, differentially expressed genes; DIS, dilated intercellular spaces; *DSG1,* desmoglein 1; EA, eosinophil abscess; EGD, esophagogastroduodenoscopy; EGIDs, eosinophilic gastrointestinal diseases; EI, esophageal inflammation; EoC, eosinophilic colitis; EoD, eosinophilic duodenitis; EoE, Eosinophilic esophagitis; EoEHSS, EoE Histology Scoring System; EoG, eosinophilic gastritis; eos/HPF, eosinophils per high power field; EREFS, Endoscopic Reference Scores; ESL, eosinophil surface layering; GSEA, gene set enrichment analysis; H&E, hematoxylin and eosin; *HDC*, histidine decarboxylase; IL, interleukin; *IL13RA2*, interleukin 13 receptor subunit alpha 2; *IL2RA*, IL-2 receptor subunit alpha; IL-5Rα, IL-5 receptor alpha; *IL7R,* IL-7 receptor; *KRT14*, keratin 14; LPF, lamina propria fibrosis; n/a, not applicable or available; NES, normalized enrichment score; *POSTN,* periostin; PPIs, proton pump inhibitors; SEA, surface epithelial alteration; *VDR*, vitamin D receptor.

## Notes

Specific author contributions: Ashley L. Pyne PhD, RDN participated in the preparation of sequencing results, analyzed data, and drafted the manuscript. Amiko M. Uchida, MD provided a critical review of the paper and manuscript revisions. Mark W. Hazel, PhD participated in planning and preparation of sequencing results, and manuscript revisions. Chris Stubben, PhD analyzed gene expression, contributed to the interpretation of results, and drafted figures. Joy W. Chang. MD, MS provided a critical review of the paper and manuscript revisions. Dominque D. Bailey, MD, MSEd provided a critical review of the paper and manuscript revisions. Nirmala Gonsalves, MD provided a critical review of the paper and manuscript revisions. Kristina Allen-Brady, PhD MSPH contributed to acquisition of sequencing results and critical revisions of the manuscript. Kathryn A. Peterson MD, MScI planned and supervised the project, reviewed and interpreted clinical care results, and drafted and revised the manuscript. Maria A. Pletneva, MD, PhD reviewed and interpreted all histologic results, and drafted and revised the manuscript. All authors approved the final version of the manuscript.

Financial support: JWC is supported by funding in part by NIH award K23DK129784. AMU is a Consortium of Eosinophilic Gastrointestinal Diseases Researcher Trainee Scholar. DDB is supported by funding in part by NIH award K08DK128603, the Robert Wood Johnson Foundation’s Harold Amos Medical Faculty Development Program, and Columbia University Digestive and Liver Disease Research Center (CU-DLDRC) grant 1P30DK132710.
