# Supplementary material for: Effect of benralizumab on histopathology and inflammatory signatures in a clinical cohort of eosinophilic esophagitis
Source: Dis Esophagus. 2024 Jul 11;38(1):doae031. doi: 10.1093/dote/doae031 (PMC11734470; doi:10.1093/dote/doae031)
Supplement: Benralizumab_and_EoE_Supplemental_Figures_doae031 [file benralizumab_and_eoe_supplemental_figures_doae031.docx]

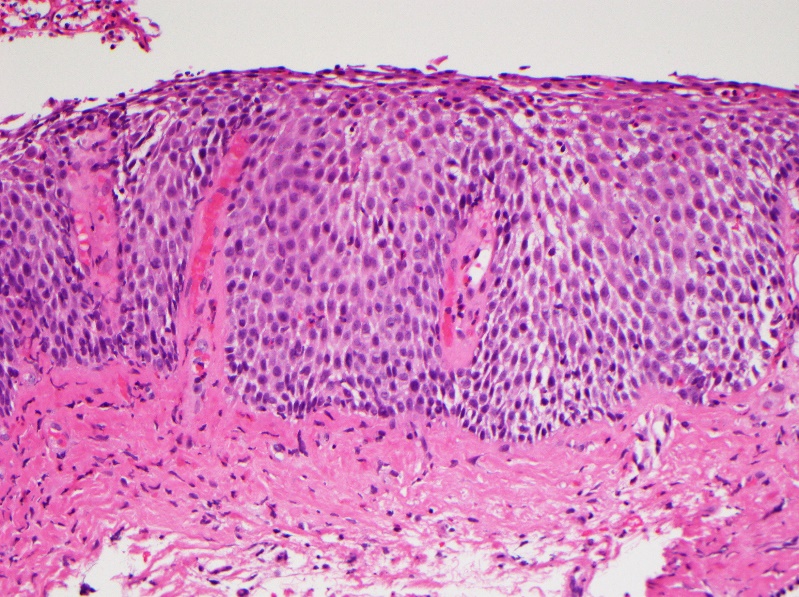

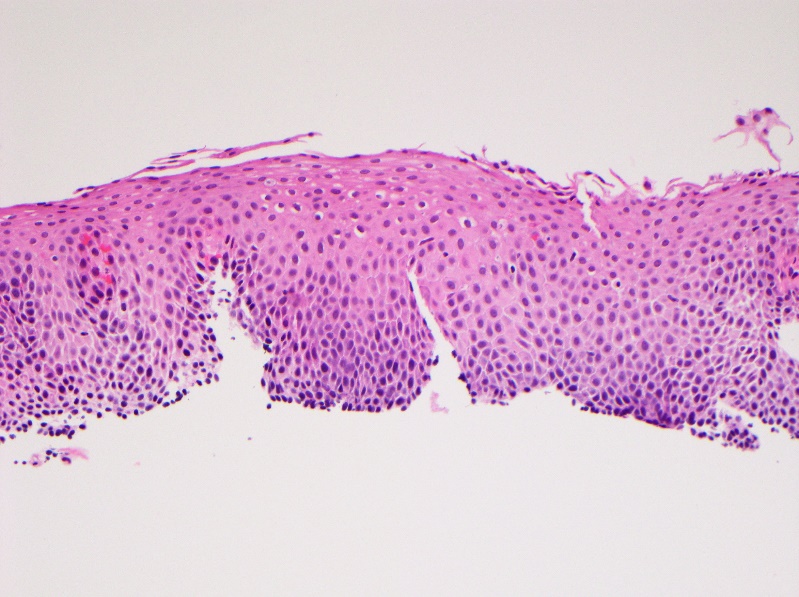

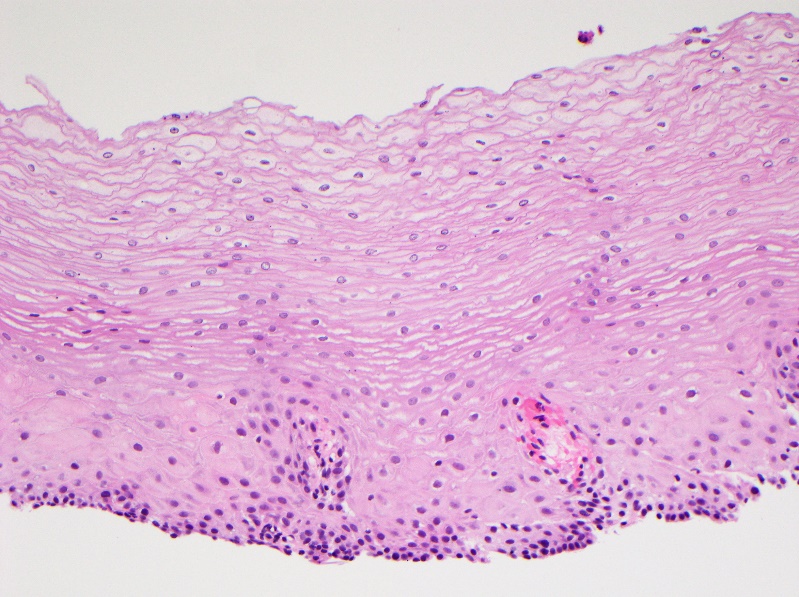

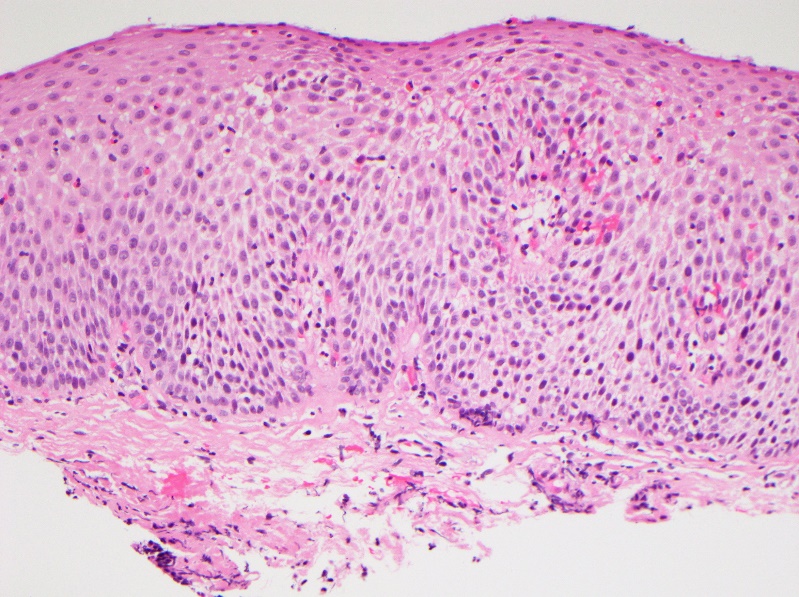


A

B

C

D

**Supplemental Figure 1**. Benralizumab treatment results in eosinophil eradication but does not ameliorate epithelial features of disease. H&E-stained sections of esophageal biopsies from patient before (A) and after benralizumab treatment (B), and patient before (C) and after diet (D) [image magnification 200x]. Single arrows denote dilated intercellular spaces. Open arrows point to eosinophils. Bars denote extent of basal zone hyperplasia. Double arrows point to surface epithelial alteration (with (A) and without (B) eosinophilic exudate). Star denotes lamina propria fibrosis.

**Supplemental Figure 2.** Eosinophils (eos/HPF) were depleted in stomach and duodenum following benralizumab (post-benra) treatment in patients with comorbidity of gastritis and duodenitis (tissue available for review for three of the four patients).

**
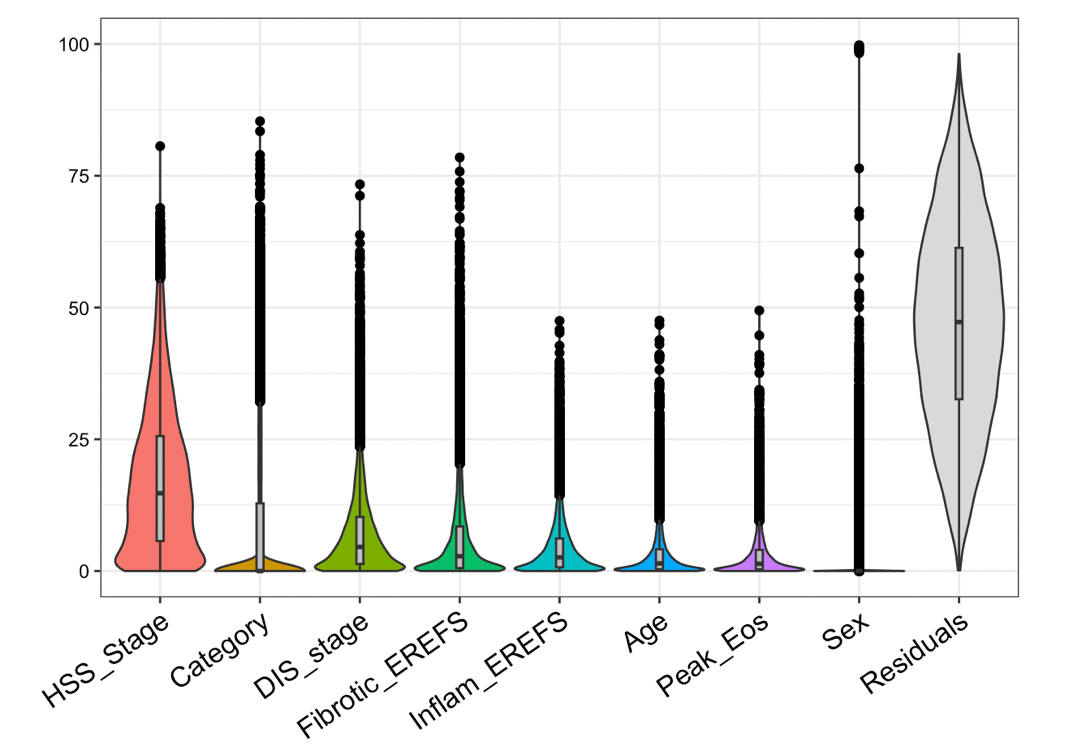
**

**A**

**B
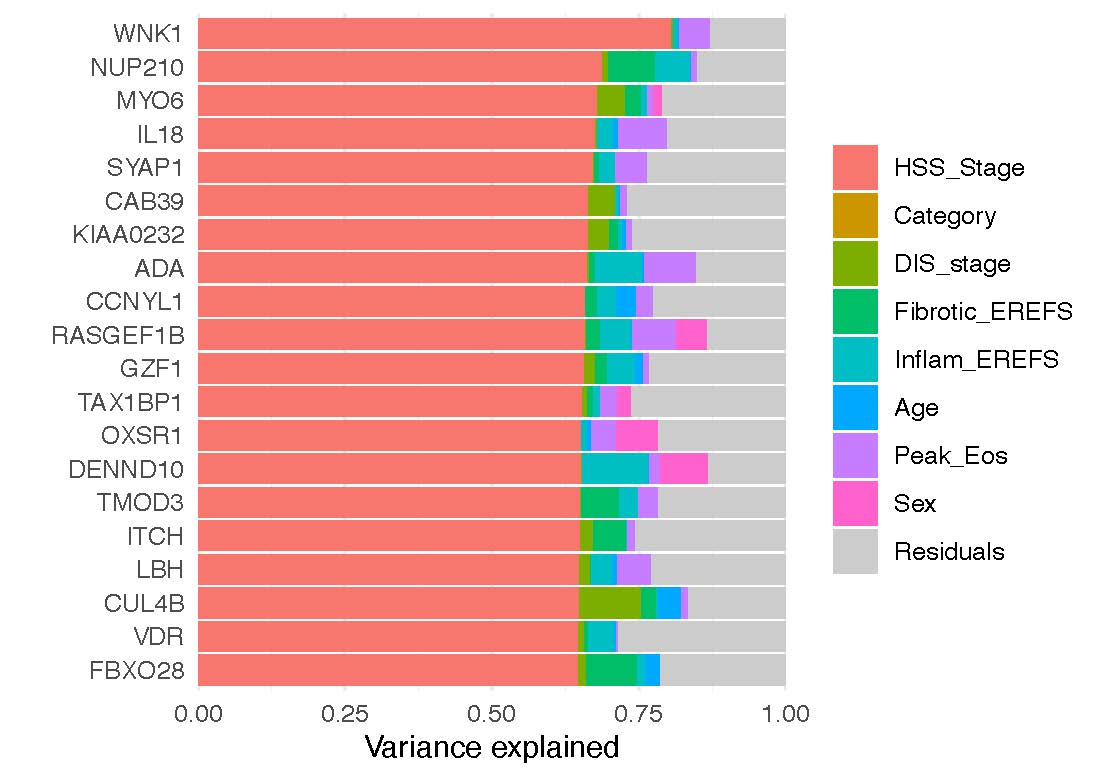
**

**Supplemental Figure 3.** The largest fraction in the variance of gene expression in benralizumab treated patients is explained by the disease severity parameter HSS stage scores (A) Violin plot of percent variation in gene expression explained by each variable in the linear mixed effects model. (B) Total variance for the top 20 genes with highest fraction of variance attributable to HSS staging. Variables included: EoEHSS stage score (HSS_Stage), Patient categories of benralizumab treated patients, active EoE, remission EoE and non-EoE controls (Category), Dilated Intercellular Space stage score (DIS_stage), summed score for fibrotic parameters of EREFS (Fibrotic_EREFS), summed score for inflammatory parameters of EREFS (inflam_EREFS), age, peak eosinophil count/HPF (Peak_Eos), sex and residual variance.
